# Qualitative longitudinal research in vocational psychology: a methodological approach to enhance the study of contemporary careers

**DOI:** 10.1007/s10775-024-09692-5

**Published:** 2024-08-09

**Authors:** J. Masdonati, C. É. Brazier, M. Kekki, M. Parmentier, B. Neale

**Affiliations:** 1 Institute of Psychology, University of Lausanne, UNIL-Mouline, 1015 Géopolis, Lausanne Switzerland; 2 Swiss National Centre of Competence in Research LIVES – Overcoming vulnerability: life course perspectives (NCCR LIVES), University of Lausanne, Lausanne, Switzerland; 3 Management School, HEC Liège, University of Liège, Liège, Belgium; 4 School of Sociology and Social Policy, University of Leeds, Leeds, UK

**Keywords:** Qualitative methods, Qualitative longitudinal research, Vocational psychology, Career development, Career transitions

## Abstract

Although temporality is pivotal to most career development processes, qualitative longitudinal research (QLR) is still rare in vocational psychology. QLR consists of following individuals over the years and exploring how they develop through time. It implies articulating themes, cases, and processes to reach an understanding of change in the making. Based on two vignettes showing how the entourage influences career change processes, we address the heuristic, praxeological, and transformative potential of using QLR in vocational psychology and, more specifically, to study career transitions. This approach also raises practical and ethical challenges that must be considered.

## Résumé

Bien que la temporalité soit essentielle à la plupart des processus de développement de carrière, la recherche qualitative longitudinale (RQL) est encore rare en psychologie de l'orientation. La RQL consiste à suivre des individus au fil des années et à explorer comment ils se développent dans le temps. Elle implique l'articulation de thèmes, de cas et de processus pour parvenir à une compréhension du changement en cours de réalisation. Basée sur deux vignettes montrant comment l'entourage influence les processus de changement de carrière, nous abordons le potentiel heuristique, praxéologique et transformateur de l'utilisation de la RQL en psychologie de l’orientation et, plus spécifiquement, pour étudier les transitions de carrière. Cette approche soulève également des défis pratiques et éthiques.

## Abstract (de)

Obwohl die Zeitlichkeit für die meisten Karriereentwicklungsprozesse von zentraler Bedeutung ist, ist qualitative Längsschnittforschung (QLR) in der Berufspsychologie noch selten. QLR besteht darin, Individuen über die Jahre zu verfolgen und zu erforschen, wie sie sich im Laufe der Zeit entwickeln. Dies impliziert die Artikulation von Themen, Fällen und Prozessen, um ein Verständnis für Veränderungen im Entstehen zu erreichen. Basierend auf zwei Vignetten, die zeigen, wie das Umfeld Karriereveränderungsprozesse beeinflusst, thematisieren wir das heuristische, praxeologische und transformative Potenzial der Verwendung von QLR in der Berufspsychologie und speziell zur Untersuchung von Karrieretransitionen. Dieser Ansatz wirft auch praktische und ethische Herausforderungen auf, die berücksichtigt werden müssen.

## Resumen

Aunque la temporalidad es fundamental para la mayoría de los procesos de desarrollo de carrera, la investigación longitudinal cualitativa (QLR) aún es rara en la psicología vocacional. QLR consiste en seguir a los individuos a lo largo de los años y explorar cómo se desarrollan con el tiempo. Implica articular temas, casos y procesos para alcanzar una comprensión del cambio en proceso. Basándonos en dos viñetas que muestran cómo el entorno influye en los procesos de cambio de carrera, abordamos el potencial heurístico, praxeológico y transformador de usar QLR en psicología vocacional y, más específicamente, para estudiar las transiciones de carrera. Este enfoque también plantea desafíos prácticos y éticos que deben considerarse.

## Introduction

Sophisticated research methodologies need to be implemented to expand our understanding of contemporary careers, which are increasingly characterized by unpredictability, challenging transitions, and complex career decision-making processes (Chudzikowski, 2012; Fouad and Bynner, 2008; Lent and Brown, 2020; Levin and Lipshits-Braziler, 2022; Sullivan and Al Ariss, 2022). While a longitudinal approach is progressively considered essential in quantitative careers studies in psychology (Akkermans et al., 2021; Rudolph, 2021), qualitative longitudinal research (QLR; Neale, 2021b) is rare, although it has been used to good effect in sociology, for example, in studies of the career trajectories of young people (e.g., Bidart et al., 2013), those in mid to later life (e.g., Hermanowicz, 2009), or people undergoing welfare to work interventions (e.g., Danneris, 2018).

In the present methodological paper, we stress the relevance and challenges of implementing QLR in vocational psychology^1^ to study lifelong career development and, more specifically, career transitions. First, we make the case for QLR in vocational psychology by stressing the temporal nature of contemporary careers, describing how research in vocational psychology traditionally addresses time, describing the main features and procedures of QLR, and highlighting its value for studying career development and transitions. Second, the potential significance of QLR is illustrated through the presentation of vignettes from two participants in a study on relational influences on involuntary career change. Third, we provide an overview of the strengths and challenges of conducting QLR in vocational psychology.

## Making the case for qualitative longitudinal research in vocational psychology

### The temporal nature of career development

Temporality “refers to the state of existing in time” (Olry-Louis et al., 2022, p. 257) and encompasses “everything that relates to time, whether it is the perception of simultaneity, succession or duration, of past/present/future, or how the individual experiences specific moments of time” (p. 258). Temporality is at the core of most career-based behaviors and is inherent in most theories of vocational psychology, such as the life-span, life-space approach to careers (Super et al., 1996), career construction theory (Savickas, 2020), and systems theory framework of career development (Patton and McMahon, 2015). More specifically, it is a key factor in career decision-making, one of the main tasks within career development, which implies a thorough self-awareness and the ability to anticipate the future and to project into possible selves (Gati and Levin, 2015; Ibarra and Petriglieri, 2010; Lent, 2013). Future temporality, as seen in the present situation, is thus of major importance.

Coping with career transitions is another crucial, inherently temporal career-based task (Olry-Louis et al., 2022; Sullivan and Al Ariss, 2022). A successful transition involves not only being able to tackle the challenges of the ongoing change but also moving on from the past and integrating a new work situation while preserving a minimum of continuity over time (Kulkarni, 2020). Thus, the identity work implemented in such a transition consists of maintaining continuity of self despite the career change, which involves articulating the future with the past (Zittoun, 2009). Coping with a transition also often involves parallel challenges in other life spheres (e.g., health and family), which have their own rhythms and may be more or less synchronized with the individual’s career (Perkins et al., 2023).

Within contemporary careers, temporality is not only central but also underlies challenging processes. Career decision-making has become an incredibly complex task, as the process of anticipating a career is hampered or even rendered irrelevant by a labor market that is constantly, unpredictably, and rapidly changing (Lent, 2013; Lent and Brown, 2020; Levin and Lipshits-Braziler, 2022). A volatile socioeconomic context also makes it difficult to cope with career transitions, which tend to become less predictable and more frequent (Chudzikowski, 2012; Sullivan and Al Ariss, 2022). For example, unexpected career transitions prevent workers from preserving self-continuity by anticipating possible selves consistent with past and present selves (Brazier et al., 2024; Conroy and O’Leary-Kelly, 2014). In addition, having to cope with repeated career transitions can jeopardize workers’ health and overall life satisfaction (Udayar et al., 2024).

### Time-sensitive research in vocational psychology

Given the temporal challenges that characterize contemporary careers, implementing time-sensitive research becomes crucial in vocational psychology (Dlouhy and Biemann, 2017). Temporality can be researched retrospectively or prospectively (Audulv et al., 2023; Neale, 2021a; Olry-Louis et al., 2022). Retrospective studies aim to understand the present situation in light of past experiences; prospective studies involve tracking the development of participants through “real” time. Both quantitative and qualitative methods can be implemented to address temporal questions, be it retrospectively or prospectively. While quantitative methods are nomothetic and pinpoint generalizable trends and causal patterns, qualitative methods are idiographic and enable researchers to delve deeper into processes, individual experiences, and subjective understandings of causality (Blustein et al., 2005; Neale, 2021a; Ponterotto, 2005).

In the field of vocational psychology, quantitative research is dominant, with longitudinal quantitative designs being increasingly prevalent in studying career transitions (Akkermans et al., 2021; Akkermans et al., 2024). Qualitative research is still marginal in the field, even if it becomes more visible (Heppner et al., 2016; Richardson et al., 2022; Stead et al., 2012). For example, in their meta-study of articles published by the *Journal of Career Development* between 2000 and 2019, Mehlhouse et al. (2023) stressed that the ratio of qualitative papers was 20%, one of the lowest among counseling journals. Moreover, existing qualitative research in vocational psychology is merely cross-sectional. For example, all the qualitative papers published in the *Journal of Career Development* and the *International Journal for Educational and Vocational Guidance* in 2023 were based on cross-sectional studies. These works provide a fine-grained picture of the vocational processes at play and support the heuristic value of an idiosyncratic approach to career development. At the same time, the sole focus on cross-sectional designs prevents us from understanding career development experiences as they unfold (George et al., 2022; Neale, 2021b).

Recent literature reviews tentatively suggest that more qualitative studies should be conducted to better grasp career decision-making (Richardson et al., 2022) and contemporary career transitions (Sullivan and Al Ariss, 2022). In particular, QLR is called upon to follow individuals throughout their career transitions (Akkermans et al., 2024; Sullivan and Al Ariss, 2022). The value of such an approach has been recognized across disciplines, with QLR now being a well-established methodology, for example, in health and nursing research (e.g., Audulv et al., 2023; Pinnock et al., 2011; SmithBattle et al., 2018).^2^ Of particular relevance are a range of studies that have explored career transitions and trajectories in disciplinary contexts other than vocational psychology. In youth research, for example, QLR has been used to trace the career trajectories of young people and their transitions from school to work (e.g., Bidart et al., 2013; Hodkinson et al., 1996), while the trajectories and changing fortunes of older workers and job seekers have been traced through time in a variety of ways. In sports psychology, Torregrosa et al. (2015) investigated athletes’ transitions to retirement by interviewing participants before and 10 years after retirement. Hermanowicz (2009) used a similar follow-up approach, whose longer-term re-study traces the careers of academic scientists over a decade of change. Keskinen et al. (2023) used a more intensive processual design over a shorter time span to understand the shifting strategies employed by older job seekers to overcome perceived ageism in the labor market. Finally, social policy researchers have used QLR to good effect to trace the changing fortunes of poorly resourced people who are subjected to increasingly punitive welfare-to-work programs (e.g., Danneris, 2018; Neary et al., 2021; Patrick, 2017) or have explored how particular groups (e.g., single mothers or young fathers) cope with the everyday challenges of balancing precarious work and experiences of poverty with their caring responsibilities (Millar, 2007; Neale and Davies, 2016). Many of these studies explore and illuminate the intersection of career trajectories with other life factors (e.g., socioeconomic, relational, geographical, and age-based) (e.g., Neale and Tarrant, 2024).

However, despite these widespread developments across disciplines, QLR is overlooked in vocational psychology. This is surprising given the temporal nature, unpredictability, and changeability of contemporary careers (Chudzikowski, 2012; Fouad and Bynner, 2008). Yet vocational psychology would benefit from implementing QLR to better understand career issues and processes and the intricate dynamics of career transitions (Akkermans et al., 2021; Sullivan and Al Ariss, 2022). Indeed, although, similar to any qualitative approach, QLR does not allow for the generalization of results, it provides a distinctive lens through which to explore individuals’ experiences of change and unveil the intrinsically subjective nature of vocational processes.

### Characteristics and analytical strategies of qualitative longitudinal research

QLR consists of analyzing qualitative data collected longitudinally. It aims “to look forward, prospectively, and backward, retrospectively, to give a detailed, processual understanding of change in the making” (Neale & Tarrant, 2024, p. 53). Most QLR involves following individuals or groups over the years and exploring how they develop through time. According to Neale (2021b, Neale and Tarrant, 2024), QLR is processual in nature and implies moving from “pictures” to “movies,” asking “how” things work or change instead of “what” works and changes. This approach allows researchers to understand how continuities and changes are negotiated and experienced and to study the fluidity of temporal processes at play (Neale, 2021a). Temporal processes and the dynamics of change can be examined either intensively (through dense data collection over relatively short periods) or extensively (through more distant data collection over more extended periods) (Audulv et al., 2023; Neale, 2021b). Whether intensive or extensive, QLR involves moving beyond comparing two (or more) snapshots in time to examine different outcomes at times A and B. Rather, it aims to understand *how* and *why* individuals move from A to B, highlighting the changes in perception and identity that accompany and intersect with concrete changes in circumstances and practices (Neale, 2021b; Zittoun, 2009). This brings new perspectives into the complexities of causal processes, revealing their relational, fluid, and multiple dimensions (Dall and Danneris, 2019; Neale, 2021a).

QLR is considered a general and flexible approach to research that can be operationalized differently depending on each study’s specific paradigm and research question (Audulv et al., 2023; Neale, 2021b). For this reason, QLR can be aligned with other qualitative approaches, such as interpretative phenomenological analysis (Farr and Nizza, 2019; McCoy, 2017) and thematic analysis (Neale, 2021b). The analytical logic that shapes QLR—from devising research questions and generating data, to data analysis and the presentation of findings—revolves around an iteration between case, thematic, and processual insights (Audulv et al., 2023; Neale, 2021b). This iteration, which recognizes the fluidity of processes, has been promoted as a central tenet of QLR methodology (Neale, 2021b). However, the way this iteration occurs is flexible across different studies. As both Neale and Audulv et al. show, how and to what extent these analytical facets are linked together in practice, what priority is accorded to them, and over what time scales vary greatly.

Taking up this theme in their scoping review of circa 300 QLR studies, Audulv et al. (2023) separate three broad modes of longitudinal analysis. The first concerns studies with a low utilization of longitudinal data. The longitudinal elements of time and change are commonly subordinated to an overly thematic analytical focus and may be lost sight of when data are analyzed and findings presented. Given these limitations, the authors suggest that such studies should be given a separate identity beyond the QLR label. The second mode refers to studies structured according to chronological (linear) time (including recurrent cross-sectional or time series studies) that enable comparison between snapshots taken at different points in time. The third is the most fully developed QLR studies, for they take a processual (through-time) approach to the analysis of QLR data and recognize that beyond a chronological reconstruction, processes are inherently fluid and unpredictable, and causality is necessarily complex (Neale, 2021a). Within this more rounded processual approach, the study of longitudinal cases, themes, and processes may be separated or combined creatively, not least through a cumulative and incremental process of knowledge building through time itself.

Beyond the prevailing structural principle for addressing time and change, any qualitative longitudinal analysis can be considered to involve four steps—the emphasis being placed on one or other of these steps depending on the type of research question: (1) case description (e.g., pen portraits or case profiles summarizing each case and how it unfolds through time), (2) within case comparison (e.g., grid analyses across time for each case exploring converging or diverging trajectories), (3) within case process tracking (e.g., mapping the processes for each case), and (4) cross-case process analysis (e.g., comparing and grouping similar configurations of processes across cases) (Brazier et al., 2023a; Neale, 2021b). Processes addressed in the third and fourth stages can be described and portrayed according to several questions (Saldaña, 2003), such as: What is constant, consistent, or recurring over time? What increases, emerges, or is cumulative over time? What diminishes, ceases, or is missing over time? What is idiosyncratic across time? How is the experience of temporality characterized? What are the rhythms and the peaks at play?

### Toward qualitative longitudinal research in vocational psychology

As we have seen, studies from disciplines other than vocational psychology confirm that QLR is a powerful way to explore career issues and dynamics. Specifically, QLR seems relevant to study life and career transitions (Treanor et al., 2021). QLR entails collecting data with people undergoing a transition to understand their lived experiences and retrospective meaning-making of what contributed to the transition. Follow-up interviews yield insights into the processual development of the transition. In addition to an objective, factual development, QLR addresses “narrative change,” that is, “the unfolding of individual stories across time” (Vogl et al., 2018, p. 178). Such interviews can also help to explore retrospectively how the transition narrative evolves through time. In this case, the focus is on “participants’ reinterpretation of experiences or feelings that they described earlier” (p. 178). Nevertheless, in vocational psychology, little qualitative research has focused on the development and progress of career transitions nor on how meaning-making evolves through time (Sullivan and Al Ariss, 2022).

More specifically, relevant longitudinal research questions around career transitions can be framed in relation to thematic, case, and processual investigation strategies (Audulv et al., 2023). For example, a longitudinal themes approach could help understand the evolving experiences of labor market integration processes by comparing young adults’ expectations of entry into the labor market with their concrete integration experience—which can be considered repeatedly during the first months or years of employment. A longitudinal case approach could consist of building a typology of career trajectories following a significant event, such as parenthood, forced inactivity, ill health, or a return to training. A longitudinal process approach could lead to the description of the process and common phases of leaving the world of work and entering retirement or unemployment. While there is no set order for building an analytical strategy, a full QLR leading to a processual analysis might take time to build, starting with a thematic analysis after the first wave, building case and cross-case analyses incrementally after each wave and culminating in a processual analysis as the fieldwork comes to an end. In this way, layers of insight are built up over time.

## An illustration: investigating career change processes

To illustrate the relevance of applying QLR in vocational psychology, we draw on an ongoing two-phase, eight-year research program on involuntary career change in Switzerland (Swiss National Science Foundation fundings 100019_192429 and 10001_227634). This program was founded on the insight that little research exists on unexpected and unintentional career transitions (Akkermans et al., 2024; Sullivan and Al Ariss, 2021) and that the rare studies on involuntary career change addressed a specific population (e.g., injured veterans, Kulkarni, 2020, or athletes, Chen and Bansal, 2022). However, involuntary career change—i.e., an unintentionally triggered move to a new occupational field—needs to be better understood, as it can prove to be a challenging career transition. Indeed, workers forced to change careers face several individual, environmental, and institutional barriers (Fouad and Bynner, 2008). Based on these observations, our research aimed to understand how involuntary career change experiences unfolded while also considering the relational influences underlying these experiences. During the first phase of the research program, over a 2-year period, we carried out three waves of interviews with three groups of workers: those who had been forced to change careers because of physical or mental health problems, unemployed people in declining occupational sectors, and migrant job seekers whose qualifications were not recognized in Switzerland.

### A cross-sectional analysis of relational influences on involuntary career change

At the end of the first wave of interviews, we conducted a series of cross-sectional analyses focusing on the interpersonal aspects of career transitions (Masdonati et al., 2022). Through thematic analysis (Braun and Clarke, 2019) of participants’ recollections of their career change process, we showed that relational influences could be divided into three sources (i.e., from personal environments, support structures, or organizations) and take three forms (i.e., positive, negative, or ambivalent). Moreover, participants retrospectively indicated that relational influences operated in distinct ways depending on whether they were leaving their former occupation, shifting from the former to a new occupation, exploring new career options, or implementing a new career plan.

Among the most salient results, we showed that relational influences can involve two forms of ambivalence (Masdonati et al., 2022). The first form is situated ambivalence and refers to the fact that the exact source of influence could both support and hinder the career change process. For example, for some career changers, institutional influences were simultaneously a barrier regarding rules rigidity and a resource, thanks to the support of committed career professionals. Conversely, ambivalence can also be temporal, referring to the insight that the exact source of influence could have changing effects over time. For example, some participants reported that their close ones initially hindered the career change process and became more supportive as the process progressed.

Nevertheless, because of the lack of longitudinal data, these retrospective perspectives did not allow us to explore the temporal ambivalence in-depth and at the moment of its operation. Moreover, while collecting data for the second and third waves of interviews, it became clear that the narratives about relational influences fluctuated, sometimes even radically changing. Longitudinal analyses seem, therefore, the best way of gaining a finer understanding of the possible shifting relational influences on the processes of involuntary career change and how they are experienced as they occur. We illustrate the potential of this type of analysis by reporting on the evolution of relational influences from the personal environment of two participants, Jean and Josefa, having both been interviewed three times over two years. The cases were consensually selected by the authors of the present paper, since they vividly illustrated distinct evolutions in relational influences. The first two authors conducted a draft temporal thematic analysis (Neale, 2021b) to identify the main processual threads of each case. Without intending to provide a comprehensive analysis of study data, these cases are designed to illustrate the relevance of collecting and analyzing longitudinal qualitative data to uncover new insights.

### The case of Jean

When we met him for the first interview, Jean was 31 years old and had learned that he could not continue being a truck driver due to an accident that prevented him from working in a seated position. With the support of public invalidity insurance, he was considering a career change toward the security sector. The striking finding regarding Jean’s relational influences is that, at the beginning of the career change process, the persons closest to him, particularly his partner, did not understand him. Jean felt he was being judged and was considered lazy in dealing with his career change. This relational tension led him to fear a break-up with his partner, to isolate himself, and not to share his difficulties with her and his close friends: “I’m not the one who’s going to spontaneously talk about it. If someone asks me, I’m happy to talk about it, but I’m not going to call up my mates and say, ‘I’ve got a training course.’ I don’t really feel like getting excited until it’s concrete.”

When we met him 1 year later for the second interview, Jean had completed a short training and found a fulfilling position as a security manager. He reported that the tensions with his close relationships, particularly his partner, eased as he implemented his career plans, “She admits that she was harsh and not fair, that now when she sees what’s happening now, how happy I am, she totally screwed up on her behavior.” His partner and friends were now emotionally supporting him in his efforts, “Once I started working, quite the opposite, it was total support.” Consequently, Jean no longer felt ashamed to share his situation with others. However, his entourage tended to attribute the success of Jean’s career change to luck and not to his efforts, which irritated him.

At the third interview, Jean’s situation had stabilized; he had obtained a full-time permanent contract in a job that suited his limitations. Generally satisfied, he was still concerned about the after-effects of his accident, which were still present, and the fear of experiencing overwork. In terms of his relationships with his close circle, the patterns identified in the second interview were further consolidated. He saw his family as an important resource and was increasingly able to talk about his career change experience. This resulted in him finding it easier to share his experiences in general and also, advocating for a better social representation of disability insurance and its beneficiaries:

Now that it’s classified, I talk about it because I’m no longer ashamed to say where I am now, I’m working for such and such, and that’s that. And if people ask me questions about what, when, and how, well, I explain that too because, once again, I want to improve the image of disability insurance.

Finally, as in the second wave of interviews, he still sometimes felt a certain judgment on the part of his entourage: “I talk about it a lot more freely; after that, I always have a bit of a twitch in my eye when people say to me: ‘ah but you were lucky then.’”

### The case of Josefa

When we interviewed Josefa for the first time, she was 35 years old, married, and mother of one child. She had arrived from Western Europe in Switzerland 7 years before and worked as a cleaner, then as a housekeeper in a private clinic and a hotel. She suffered burnout and had chronic health issues preventing her from continuing to work in her field. Having been made redundant, she was unemployed and aspired to retrain as an administrative assistant. Her husband was supportive and understanding, telling her that work is less important than health. She also used to talk a lot about her situation with her mother. She reported her son wanting to help her, “They completely understand the difficulties I have on a day-to-day basis with my health, and if I find a job that I like and that I can do without any problems, that would be a relief for them too.” Feeling support was important to her since it helped “to cope with the ups and downs of life.”

During the second interview, Josefa reported that she was neither officially unemployed nor on disability insurance and that her doctor recommended that she apply for disability funding. She mentioned her loneliness and her sadness for not being able to have normal family activities, which her son reminded her of repeatedly during the past year. She experienced both being a burden and feeling guilty for not having a salary, “If I earned a little money for the house, I’d feel more useful too, not to overload my husband, for example with, only with his salary.” She no longer spoke to her family, except to her husband, because they were not able to understand her pain and her challenging experience: “Even we don’t understand our bodies sometimes. How are people going to understand? How if they don’t have it, they’ve never experienced it in their lives?” On the other hand, she had recently joined a peer support group, where she found compassion and mutual support.

At time 3, she was still awaiting a disability insurance decision and could not engage in any professional activities for the past year. She found it very hard to cope with being out of work. However, she described being in an emancipatory process in which she could redefine her needs and limits and assert herself. As a consequence, she was less reluctant to talk about health issues and their impacts: “I used to feel a bit ashamed of explaining to people that I had a few limits, or whatever. And now, for example, I was at the wedding and said, ‘I don’t feel well’ if I didn’t feel well.” She also became very involved in the peer support group and hoped to raise awareness of the issues.

### Toward a longitudinal understanding of relational influences on career change

These vignettes can result in different observations, depending on whether researchers focus their analytical attention primarily on themes, cases, or processes. A thematic longitudinal focus means pinpointing key themes within both vignettes and their evolution. This would lead to the statement, for example, that the sources and forms of Jean’s and Josefa’s relational influences were diversified. In both cases, the partner seemed to play a pivotal and enduring role, whereas other sources of influence from the personal environment were more volatile. While the partners’ role remained central throughout the process, the form of their influence varied through time: they could be perceived as much support as an obstacle (notably by making judgments or pressuring the career changers). The experience of the change process thus appears to be firmly and constantly dependent on the validation from partners.

A case-centered longitudinal focus would prioritize the comparison of the two vignettes. At Time 1, Josefa was better supported than Jean, who experienced his personal environment as more of a barrier. However, their situation evolved distinctively: Josefa felt her family began to fail to understand her struggle and pain. In contrast, Jean felt people close to him increasingly supported him while having been “suspicious” at time 1. The unfolding of the career change situation and the passing of time seem to have operated differently. Time “worked in Jean’s favor,” allowing those around him to gradually understand the complexity of his career change process. This gradual awareness was doubtless facilitated by the fact that Jean could activate himself and find a satisfying new occupation. On the contrary, time “worked against” Josefa. For example, her family was initially empathetic to her difficulty in coping with multiple health issues and changing careers. Yet, this empathy seems to fade as time goes by and Josefa does not integrate back into the labor market. This progressive incomprehension can be attributed to her stagnant career change process, where nothing changes for 2 years. Thus, in Josefa’s case, relational influences move from an understanding attitude to one of incomprehension, leading her to reduce the circle of people she could open up to and seek authentic support elsewhere (i.e., in a group of peers). Time seems to have had a progressive filtering-displacement effect, while in Jean’s case, time had a settling effect over the three study waves.

Finally, a process-oriented focus would indicate that, beyond these distinct developments, common processes appear to characterize the relational dynamics surrounding the unfolding of Jean and Josefa’s career change. In both cases, it seems that the initial reactions of their entourage did not completely fade with time, as if there were some sort of residual relational influences. In Josefa’s case, this can be seen in her recognition of her husband’s central and constant supportive role; in Jean’s case, there is still a hint of judgment in his perception of how his entourage sees his career instability over the years. Moreover, although each in their way and rhythm, Jean and Josefa have gone from an initial period of suffering, reflected in self-isolation and feelings of shame, to a phase of self-affirmation and empowerment. Indeed, 2 years after initiating the career change process, Jean advocated the importance of disability insurance support, while Josefa took an increasingly leading role in her peer support group.

Overall, this illustration shows that the added value of a qualitative longitudinal analysis lies in its potential for understanding how the impact of others on involuntary career change unfolds and through which underlying processes. These findings, if echoed in the narratives of other participants, could indicate the usefulness of the support of personal entourage in implementing a new career plan as a source of reinforcement for a process already oriented toward resolving the career change. Conversely, the role of this entourage has its limits and seems more fragile when the person is stuck in a liminal state and does not give the impression of advancing in the process. For Jean, this period was short but left behind some frustrations; for Josefa, this period was prolonged, leading her to look elsewhere for the support she lacked from her entourage.

This observation on the impact of others on involuntary career changes would have relevant implications for research in vocational psychology and career guidance and counseling practices. Implications for research would consist, for example, of stressing that the entourage of adults in transition can be affected by their transitional journey and that its influence fluctuates depending on the evolution and length of the career change process. Such findings would provide a more dynamic understanding of the relational and interpersonal elements that impact career transitions. In terms of implications for practice, these results would suggest the importance of providing institutional support when the personal environment may be less helpful, for example, in the complex and prolonged process of grieving the loss of one’s former occupation, finding and implementing a new career plan. Group interventions that include the entourage and aim to consider its point of view and raise awareness of its key role would also be practical implications arising from these results.

## Potentials and challenges of implementing qualitative longitudinal research in vocational psychology

In addition to its relevance to address temporal processes in vocational psychology, QLR has several distinctive strengths that make it a methodological approach with significant potential for the study of career transitions. However, the implementation of QLR in vocational psychology is not without its drawbacks and challenges.

### Heuristic, practical, and transformative strengths

Implementing QLR would be beneficial for at least three reasons, resulting from three forms of strengths of this methodological approach: heuristic, practical, and transformative. Concerning the heuristic power of QLR, the illustration of the fluctuating influences of the entourage on Jean’s and Josefa’s career transition process is just one example of the spectrum of potential new perspectives on vocational issues that emanate from QLR. Indeed, QLR provides a subtle understanding of the processual and dynamic features of a career transition (Akkermans et al., 2021; George et al., 2022; Sullivan and Al Ariss, 2022), captures the salience of temporality and shows how narratives about the experience of change evolve—or not—over time (Brazier et al., 2023a; Neale and Davies, 2016). As a result, QLR can provide a more accurate picture of what transitions mean and how they unfold. In this sense, it has a double complementarity with prevailing methodologies in the field. First, it adds a longitudinal dimension to cross-sectional qualitative research and complements it by integrating the question of time and change into the analysis of career experiences. The illustration presented above on the unfolding of relational influences on involuntary career change is an example of such a contribution. Second, QLR adds a qualitative dimension to longitudinal quantitative research with significant explanatory power; it enables an idiosyncratic and granular understanding of what may explain general trends within career development (Akkermans et al., 2024). For example, the results of person-centered research on career paths (e.g., Udayar et al., 2024) could be enhanced with qualitative data within a mixed-method study to understand how people experience different types of career trajectories.

As for the practical power of QLR, a plethora of implications for career guidance and counseling could naturally emerge from qualitative longitudinal studies. Based on the Audulv et al. (2023) typology, the results of a QLR prioritizing a themes approach may serve to identify the salient areas of a transition experience on which it is most important to concentrate support. Findings from a case approach of QLR would enable support to be tailored to the types of career trajectories counselees undertake. Studies prioritizing a process approach would help identify the most appropriate moments for counseling and yield rich insights into how lived experiences of career transitions mesh with organizational and institutional processes. Overall, the fact that QLR shows that career transitions take time to unravel and underpin demanding experiences over time is a strong argument for advocating long-term career interventions.

Concerning the transformative power of QLR, engaging in such research is already transformative for participants (Thomson and Holland, 2003). It is acknowledged that participants in qualitative studies gain from sharing their experience with a nonjudgmental interviewer interested in their subjective journey without any material or social desirability concerns. In this regard, it is worth considering a question raised by Birch and Millar (2000), “Can the invitation to narrate past and present experiences, together with future hopes, avoid offering potential therapeutic opportunities?” (p. 189). This opportunity for narration and self-reflexivity is even stronger in QLR because the same researcher usually conducts several interviews, and interviewers integrate past interview contents into new ones (Thomson and Holland, 2003). Consequently, in these settings, a bond of trust can grow over time, and the researcher might endorse the dual role of researcher *and* career counselor—as suggested by Fleet et al. (2016) for research on clinical psychology interventions. These conditions eventually facilitate awareness and new insights into the transition challenges and create a safe space for repeated shared moments that can transform a person’s relationship with their transitional experience. Such scenarios are corroborated by contemporary research and approaches that stress the relevance of narrative interventions in career guidance and counseling (Rossier et al., 2021; Savickas, 2019).

### The challenges of qualitative longitudinal research in vocational psychology

According to Neale (2021b), beyond its many strengths, conducting QLR also involves several challenges. First, efforts must be made to maintain contact and a bond with participants to ensure a high participation rate over time (Solomon et al., 2020). Second, analyzing data over several points in time, articulating cases, themes, and processes, and addressing evolving research questions turns out to be complex and calls for iterative adjustments of analysis strategies (Neale and Tarrant, 2024; Vogl et al., 2018). Third, QLR implies a longitudinal ethic, consisting of establishing researcher–participant reciprocity, maintaining professional boundaries, and designing an ethical closure for the study. Fourth, since it extends over time, QLR requires more resources than cross-sectional studies (Vogl et al., 2018). Fifth, the risk of data overload (Saldaña, 2003) and extended data collection require careful design of data management to facilitate data comparisons and connections through time so that researchers have time to conduct analyses between waves (Solomon et al., 2020). Sixth, there is a risk that the results will come out too late to address the original research or social problem. While the last three challenges relate similarly to QLR in vocational psychology and QLR in any other field, the first three challenges underpin additional issues specific to the field, calling for targeted methodological strategies.

First, maintaining a high participation rate over time can be particularly difficult when studying career transitions. Indeed, such transitions sometimes involve a change of living place and contact details (phone number or e-mail). Moreover, transition processes can be demanding, which can demotivate participants to make time for long-lasting interviews. Self-selection processes can also be insidious (Thomson and Holland, 2003), with participants who have difficulty managing their transition possibly tempted to withdraw from the study, in contrast to those with a smooth transition path. This entails the risk of accessing only successful stories and thus gaining a partial picture of the issues involved in a career transition. A range of strategies can be considered to reduce the risk of study withdrawal, such as engaging in systematic debriefing with participants at the end of each interview, being attentive and sensitive to participants’ emotional states and maintaining contact with them between waves of data collection (e.g., by sharing interim reports, cf. Brazier et al., 2023b). Regarding self-selection, ensuring transparency about the project’s aims and being explicit about the interest in accessing any transitional experience, whether successful or not, is paramount. The therapeutic value of QLR fieldwork (Thomson and Holland, 2003) can also encourage enduring commitments from the most vulnerable participants. Involvement in the research may give them a voice and a sense of usefulness and represent relief from loneliness.

In addition, having to iteratively adjust the analytical strategy in order to address evolving research questions is another major challenge of QLR in vocational psychology because of a scientific context where qualitative researchers are often expected to rely on preestablished templates.^3^ Justifying analytical flexibility can, therefore, prove complex in a field that is not necessarily accustomed to such an approach. In fact, the challenge of moving away from “rigid sets of procedures” is not specific to qualitative research in vocational psychology but concerns psychology in general (Levitt et al., 2017, p. 6), as well as nursing (SmithBattle et al., 2018) and rehabilitation studies (Solomon et al., 2020). Demonstrating the relevance of methodological and analytical flexibility is essential to overcome this risk. The recent articles by Pratt et al. (2022) and Richardson et al. (2022) supporting the value of “methodological bricolage” in guaranteeing trustworthiness for qualitative organizational studies and careers research are exemplary in this sense.

Finally, the longitudinal ethic for QLR in vocational psychology involves at least two specific features, the first being the threat to confidentiality. Indeed, access to potential participants in a vocational psychology study is often gained through public or para-public institutions offering diverse forms of support to the target population. In exchange, this usually involves sharing results with these institutions (e.g., Brazier et al., 2023b). In these exchanges, finding the right balance between reporting results that are embodied enough to be meaningful to professionals but sufficiently anonymized to prevent participants from being identified can be problematic. To ensure that participants feel free to share their experiences without fearing repercussions on the support they receive, particular care must be taken when disseminating QLR results. The second ethical challenge that seems salient for QLR in vocational psychology refers to the blurred boundaries between the roles of researcher and counselor. As researchers in vocational psychology are often also trained in career guidance and counseling, they may be tempted to “switch hats” when confronted with accounts of difficult experiences and engage in counseling to support participants needing help. If, as several qualitative researchers maintain (e.g., Birch and Miller, 2000; Fleet et al., 2016), playing a dual role is not problematic in itself, it is crucial to establish precise rules to gather insightful data while promoting participants’ wellbeing (e.g., Thomson and Holland, 2003). For example, role changes during the interview should be made explicit with the participants. Discussions within the research team are also needed to ensure that how these two roles are understood is appropriate and adjusted on a case-by-case basis and to define the limits beyond which it is advisable to refer to other professionals (e.g., psychotherapists or social workers). As with any qualitative interview with vulnerable populations, the goal is to prevent ethical tensions by establishing and maintaining a “just right” relationship in an in-between position on a continuum ranging from under-rapport to over-rapport (Schmid et al., 2024).

## Conclusions

Longitudinal qualitative research has great promise for better understanding career development and vocational behavior in a context of multiform, changing, and increasingly unpredictable careers. This methodological approach complements longitudinal quantitative research and cross-sectional qualitative research. However, several major issues are associated with its implementation, including a need to consolidate the relevance of qualitative research in the field and a more flexible approach to the rigor criteria for such studies. This suggests a paradigmatic shift in approach to research in vocational psychology. The reflections proposed in this paper are intended to accelerate this shift, which is now essential in respect of the complexity and vulnerability of contemporary careers.

## Footnotes

1 In line with Blustein et al. (2019), we define vocational psychology as “the scholarly study of work or career-based behavior and development across the life span” (p. 170).

2 For an overview of interdisciplinary and international developments, see Neale (2021b).

3 Templates refer to “standardized ways of conducting research that are used as formulas for shaping the methods themselves, especially data collection and analysis.” (Pratt et al., 2022, p. 212).
